# How lay health workers tailor in effective health behaviour change interventions: a protocol for a systematic review

**DOI:** 10.1186/s13643-016-0271-z

**Published:** 2016-06-16

**Authors:** Faith Hodgins, Wendy Gnich, Alastair J. Ross, Andrea Sherriff, Heather Worlledge-Andrew

**Affiliations:** University of Glasgow Dental Hospital and School, Glasgow, UK; University of Glasgow Library, Glasgow, UK; Glasgow Dental Hospital and School, Community Oral Health, 387 Sauchiehall Street, Glasgow, G2 3JZ UK

**Keywords:** Lay health worker, Health intervention, Health promotion, Hard to reach, Health inequalities, Tailoring

## Abstract

**Background:**

Lay health workers (LHWs) are utilised as a channel of delivery in many health interventions. While they have no formal professional training related to their role, they utilise their connections with the target group or community in order to reach individuals who would not normally readily engage with health services. Lay health worker programmes are often based on psychological theories of behaviour change that point to ‘tailoring to individuals’ needs or characteristics’ as key to success. Although lay health workers have been shown to be effective in many contexts, there is, as yet, little clarity when it comes to how LHWs assess individuals’ needs in order to tailor their interventions. This study aims to develop a better understanding of the effective implementation of tailoring in lay health worker interventions by appraising evidence and synthesising studies that report evaluations of tailored interventions.

**Method:**

Health and psychology electronic databases (EMBASE, CINAHL, MEDLINE and PsycINFO) will be searched. Reference lists of included studies will also be searched. For articles that are deemed to be potentially relevant, we will employ a ‘cluster searching’ technique in order to identify all published papers related to a relevant intervention. Cluster searching will be undertaken in an effort to maximise the breadth and depth of description of the intervention. Quantitative studies will be assessed using the *Quality Assessment Tool for Quantitative Studies*, developed by the Effective Public Health Practice Project, ON, Canada. Qualitative studies will be assessed using the Critical Appraisal Skills Programme (CASP) checklist for qualitative research. Sythesising the data will enable the development of a taxonomy of strategies for the criteria used for individual assessment of recipients’ needs and the ways in which messages or actions are tailored to these individual criteria by LHWs.

**Discussion:**

This systematic review focuses specifically on how health promotion and support is individually tailored in effective programmes by LHWs. This study will be of value to those involved in the design and implementation of interventions that utilise a LHW.

**Systematic review registration:**

PROSPERO CRD42015030071

**Electronic supplementary material:**

The online version of this article (doi:10.1186/s13643-016-0271-z) contains supplementary material, which is available to authorized users.

## Background

### Defining the term ‘lay health worker’

Lay health workers (LHWs) can form part of interventions that aim to serve ‘hard-to-reach’ individuals and communities. While LHWs are aligned to some extent to institutional health services and are supported by health professionals to deliver health promotion information and activities, they are different from other health workers due to their lack of formal professional training and their potential to have a shared background with the intervention target group or community.

Although LHWs may be recruited for a supportive role, within a health context, their aim is usually to bring about some form of health behaviour change or change in health outcome. Health behaviours include any activity undertaken for the purpose of preventing or detecting disease or for improving health and well-being [1]. Health behaviour change interventions can be defined as ‘coordinated sets of activities designed to change specified behaviour patterns’ [2].

There are many reasons for using LHWs as the channel for intervention delivery. LHWs are usually recruited because they are immersed in, or familiar with, the target community or group; therefore, they are in an optimal position to identify individuals who would benefit. This is especially useful when the intervention targets groups that are normally suspicious of, or uncomfortable with, outsiders (e.g. migrant farm workers in the USA [3] or sex workers [4]). Often, working with many hard-to-reach groups requires interventionists to meet with potential recipients in a natural setting (i.e. not clinical) and to be flexible about working hours. Furthermore, LHWs can have a shared background and ‘an understanding of a community’s cultural context for health and illness as well as its history of interactions with the service delivery system’ [5]. LHWs are, therefore, well-placed to provide individually tailored support and facilitate engagement with services [6].

### Definition of tailoring

Within a health promotion context, tailoring has been defined as:

Any combination of information or change strategies intended to reach one specific person, based on characteristics that are unique to that person, related to the outcome of interest, and have been derived from an individual assessment [7]; and,

Creating communications in which information about a given individual is used to determine what specific content he or she will receive, the contexts or frames surrounding the content, by whom it will be presented and even through which channels it will be delivered [8].

These definitions suggest that, in order to tailor an intervention to an individual’s needs, one would need to conduct some form of individual assessment of a person’s characteristics and circumstances and, subsequently, adapt the intervention delivery according to this information.

### Evidence for the effectiveness of tailored interventions and LHW interventions

A number of systematic reviews and meta-analyses have provided evidence to suggest that tailored interventions are moderately more effective than non-tailored interventions; however, the conclusions that can be drawn from the data are limited. In a systematic review of tailored interventions for smoking, there was evidence that tailored materials were more effective than no materials and non-tailored materials (RR 1.28; 95 % CI = [1.18, 1.37]) [9]. In a review of tailored interventions for physical activity and dietary behaviours, 7 out of 12 studies reported significant changes in health behaviours [10]. A meta-analysis of interventions for dietary behaviours found that tailored interventions led to individuals consuming significantly more servings of fruit and vegetables per day (weighted mean difference = 0.35; CI = [0.19, 0.52], *p* = <0.0001) and receive lower percentages of energy from fat (weighted mean difference = −2.20 %; CI = [−2.97, −1.43], *p* = <0.0001) than generic interventions [11]. These reviews were limited in the conclusions that could be drawn regarding the features of tailoring (i.e. the participant-specific variables used to inform the individualised intervention delivery; the channel, format or ‘dosage’ of tailoring; or the theory underpinning the tailored approach) that are most effective in inciting behaviour change, as this information is often not reported in sufficient detail in individual studies.

A meta-analysis of six randomised controlled trials [12] where pooled data from studies where tailored information delivered face-to-face with participants was compared with either usual care, generic health promotion or tailored print materials, which showed an overall positive effect on health behaviour using face-to-face delivery of tailored information (pooled standardised mean difference = 0.487; 95 % CI [0.02, 0.96], *p* = 0.04). Only two of the studies were deemed to have included ‘specific accounts’ of how theory translated to action. This is also the case across many health domains. For example, in the smoking cessation literature (an area of health behaviour change research that is in many other aspects well-developed), Yuan et al. [13] have commented that ‘there is a noticeable gap in the literature regarding strategies and effectiveness of tailored face-to-face tobacco cessation interventions’.

Similarly, there are gaps in the literature relating to the features of LHW interventions that contribute to effectiveness. An updated Cochrane review concluded that LHWs, when compared to usual care, have been effective in bringing about a range of positive health or health behaviour changes in communities in many different countries; however, the underlying reasons for why lay health workers may have been effective in these cases have not yet been explored [14]. The task of understanding the essential mechanisms at work within LHW interventions is complicated by the fact that the literature on such interventions provides only a partial account of the specific strategies that may be driving effectiveness. A systematic review of interventions to improve diabetes care found that the features associated with positive programme outcomes included delivery by a lay health worker; cultural tailoring; individualised assessment; delivering the intervention according to tailoring algorithms; and providing individualised feedback [15]. This review is limited to the management of diabetes, however, and does not examine the features of tailoring implemented within LHW interventions for prevention of disease.

After extensive literature searches, we have found no current literature that synthesises and systematically explores the ways in which LHWs implement the assessment of individuals’ needs and how they tailor health messages and support across programmes. This exploration and synthesis of the content of LHW interventions and the application of health behaviour change theories in effective interventions is necessary if the mechanisms for LHW effectiveness are ever to be better understood. This review will focus specifically on the content of LHW interventions that relates to tailoring the intervention to individuals’ needs and the features of such tailoring that are associated with effectiveness of the intervention.

## Aims/objectives

The aim of this systematic review is to synthesise the existing literature on the implementation of tailoring in LHW interventions; therefore, developing a better understanding of ‘what works’ in order to inform the implementation of tailoring in future LHW interventions.

Specific objectives for this systematic review are:

1. To examine the theoretical basis for tailoring in lay health worker interventions
2. To develop a taxonomy of the variables or constructs used for individual assessment of recipients’ needs
3. To develop a taxonomy of the ways in which messages or actions are tailored by lay health workers
4. To explore how support (i.e. appropriate messages or actions) is matched to assessed needs
5. To examine the evidence for the effectiveness of approaches to tailoring in lay health worker interventions

The ultimate aim of this review is to provide evidence of the use of tailoring strategies implemented in effective interventions. This will aid the development of interventions that are optimally tailored to recipients’ needs. The findings will be disseminated widely via the Executive Committee of the Childsmile programme (a national intervention in Scotland which aims to reduce inequalities in oral health and ensure access to dental services for every child). Additionally, the findings will be submitted to a relevant peer-reviewed journal and submitted as part of a thesis for a PhD degree.

## Method

We will undertake a systematic review of peer-reviewed programme evaluations where LHWs have delivered health behaviour change interventions. The PRISMA-P guidelines for systematic review protocols have been followed for developing this protocol [16]. A checklist is provided in Additional file 1. We were also guided by similar reviews (e.g. [17, 18]).

### Eligibility criteria

Peer-reviewed studies that report an evaluation of a health behaviour change intervention will be included in this systematic review. The included studies will comprise of interventions where a LHW (or multiple LHWs) is the key individual delivering the intervention. The study may be included if an individual is delivering the intervention to their own family but only when this is part of delivering it to a wider network (friends/colleagues) or community. The interventions may be delivered to children, or adults, or delivered to parents as a strategy to change child health behaviour. Individual and group interventions are included where there is evidence that an individual assessment of needs/characteristics has taken place. The intervention must allow for two-way communication between an individual and a LHW; therefore, face-to-face and telephone interventions are included and email, forum and text messaging interventions may be included if there is an exchange (back and forth) between the individual and a LHW. Interventions taking place in all contexts and settings will be considered for inclusion. The study must provide evidence that the intervention delivered is tailored, that is, one or all of the content, contexts or frames, and channels of delivery must be based on an individual assessment (formal or informal) of a person’s needs or characteristics. The outcome of the intervention must be a change in health behaviour. This may be the secondary or tertiary outcome where, for example, the primary outcome is a change in health status/physiological measurement. Studies where the intervention focuses solely on disease management (e.g. diabetes management) are excluded.

Studies reporting interventions conducted in developed countries similar to the UK context will be included (i.e. Western Europe, North America, Australia and New Zealand). Language will be restricted to English. There will be no date restrictions. Quantitative studies (e.g. randomised controlled trials and cohort studies) will be included along with qualitative studies where service users self-report behaviour change as an outcome of the intervention. A table of the inclusion and exclusion criteria is provided in Additional file 2.

### Information sources

A dedicated Science and Engineering and Medical, Veterinary and Life Sciences University Librarian (HW-A) has helped to identify health and psychology electronic databases through which the relevant studies are highly likely to be sourced. EMBASE, CINAHL, MEDLINE and PsycINFO will be searched. Reference lists of a number of reviews in the area of LHW interventions will be examined. Articles that are deemed to be potentially relevant will be included. We will employ a ‘cluster searching’ technique (explained below) in order to identify all published papers related to a relevant intervention.

### Search strategy

The search terms were developed from scoping the LHW literature and from MeSH subject headings. Search terms used in similar reviews (e.g. [12, 14]) were examined and used in a trial search. Key papers were identified through this search. Key terms, related to our inclusion criteria, used in the titles and abstracts of these papers were mapped in order to produce the minimum number of search terms required to retrieve the maximum number of relevant articles. Key terms related to our inclusion criteria included lay health worker (e.g. community health worker, health trainer), tailoring (e.g. individualise, personalise) and terms related to the kinds of activities LHWs undertake in tailored programmes, such as gaining access to hard-to-reach individuals (e.g. marginalised) and home visiting (e.g. home visit). Other key terms included health behaviour change (e.g. health promotion, behaviour change) and terms related to programme evaluation (e.g. treatment outcome, service evaluation). Also key to our inclusion criteria is that the studies originate from developed countries similar to the UK context. In *Ovid MEDLINE* and *EMBASE*, MeSH terms will be used to limit the search to Europe, North America, Australia and New Zealand. In PsycINFO and CINAHL, the search will be limited to these countries by including all variants in the search terms. Boolean operators (AND, OR, proximity) will be used to construct and refine the search.

An example of the *Ovid EMBASE* search strategy is provided in Additional file 2. The EMBASE search strategy will be adapted for the other databases.

Once individual studies have been identified, we will employ a ‘cluster searching’ [18] technique. Cluster searching refers to ‘any systematic attempt, using a variety of search techniques to identify papers or other research outputs that relate to a single study’ [18]. We will do this in an effort to maximise the breadth and depth of the qualitative description of the implementation of the intervention as well as insuring we have all available peer-reviewed literature relating to the effectiveness of the programme. Cluster searching will be carried out by checking the reference list of the key paper for ‘companion studies’, checking electronic databases for more recent references that cite the key paper, looking up the corresponding author’s more recent publications and a general Google search (Google Inc. Menlo Park, CA, USA) of the intervention and corresponding author, using a computer based in a medical sciences building on the university campus.

### Data management and selection process

Records from all searches will be imported into *EndNote* software. The records from the different databases will be combined and duplicates will be removed. Three researchers (AR, WG and FH) will independently review titles and abstracts in *EndNote* in relation to the inclusion/exclusion criteria (see Additional file 3). Papers will be included or excluded at this stage on the basis of a majority consensus, followed by discussion on disagreements.

Full text copies of the papers will then be obtained in order to assess eligibility for inclusion. At this stage, all studies will be checked to ensure that there is sufficient content reported related to ‘tailoring’. An iterative approach was used to develop the criteria for ‘sufficient content’. Calibration will be carried out amongst the review team. A study will be deemed to have sufficient content if the authors have described either a formal assessment of individuals’ needs and/or characteristics or have described how the intervention was adapted based on needs/characteristics informally gathered, or intuitively perceived, by the LHWs. If we are still unable to classify the study as tailored or not tailored, we will search for further study information (such as a website) online, search for programme process evaluations and, as a last resort, contact the corresponding author for more information.

### Data extraction

Data will be extracted from the clusters of literature related to each intervention. A draft data extraction form (FH) has been developed and piloted (see Additional file 4). Earlier versions of the extraction form were independently piloted and amendments were made. The categories in the final form include details of the design of each study conducted, the intervention, the variables/constructs used for individual assessment, the theoretical foundation for the tailoring that was implemented and how this theory (or the’ idea’ of tailoring, if no theory has been stated) was put into action considering the needs/characteristics of individuals. The recommended reporting standards for studies of tailored interventions [19] have been adapted to provide the structure for the extraction form.

There will be one team member (FH) responsible for initially extracting the data. Each of the other team members (AL, AS and WG) will be assigned a group of articles for independent extraction. The process will be iterative as AL, AS and WG will review the data extracted by FH and, after discussion, revise the extraction form to ensure all relevant data has been captured and in a consistent format and level of detail.

### Quality assessment

An assessment of the risk of bias of the included studies will be carried out collaboratively by two researchers (FH + 1 of AL/AS/WG) with discrepancies resolved through discussion. In the case of each cluster of papers, it is the papers reporting the outcome of the intervention (i.e. the effectiveness of the intervention) that will be quality assessed.

Quantitative studies will be assessed using the *Quality Assessment Tool for Quantitative Studies*, developed by the Effective Public Health Practice Project, ON, Canada [20], which has been used in a similar review [17] and generates a strong, moderate or weak quality rating. The advantage of this tool is that is can be used to assess quality across many study designs (e.g. randomised controlled trials or cohort studies). The tool allows assessment of: selection bias; study design; identified confounders; blinding; data collection methods; withdrawals/drop-outs; intervention integrity; and whether the statistical analysis was appropriate to the question. We will consider the risk of bias when assessing the overall quality of the body of evidence.

Qualitative studies will be assessed using the Critical Appraisal Skills Programme (CASP) checklist for qualitative research [21], which is one of the tools recommended by the Cochrane Qualitative Research Methods Group [22]. This assesses first whether there is a clear statement of the aims of the research and whether a qualitative approach was appropriate. The qualitative studies will be graded as weak if they do not ‘pass’ these two ‘screening questions’. They will then be graded as moderate/strong quality on the remaining questions: selection bias; study design; identified confounders; blinding; data collection methods; withdrawals/drop-outs; intervention integrity; and whether the analysis was appropriate to the question. For qualitative studies, the following aspects will be assessed: whether there was a clear statement of the aims of the research; study design and rationale; the appropriateness of recruitment and data collection, considering the aims; whether the relationship between the research and the participants was adequately considered; ethical issues; rigour of the analysis; whether there is a clear statement of the findings; and the value of the research.

### Data synthesis

The final stage will be a data synthesis and analysis with the aim of establishing the strategies used to tailor in effective programmes. Due to the likely heterogeneity of the studies, we will embark on a narrative synthesis of the extracted data. Similar approaches to data synthesis are being carried out in other systematic reviews (e.g. [23, 24]). Data will be grouped and reported in relation to the quality assessment, in order to illustrate the strategies used to translate theory into practice in those studies with the strongest evidence for intervention effectiveness. Data will also be grouped and reported in relation to the target health behaviour. In addition, we will identify any recurrent issue with reporting, with reference to the recommended reporting standards for studies of tailored interventions [19].

From the thematic analysis of the data, we will develop a taxonomy of the variables or constructs used for individual assessment of recipients’ needs, matched to a taxonomy of the ways in which messages or actions are tailored to these individual criteria by LHWs.

## Discussion

Tailored interventions utilising LHWs are being employed to serve hard-to-reach individuals and communities. While these programmes are often based on theories of behaviour change that centre on tailoring the intervention to individuals needs or characteristics, little is known about how this translates into practice across effective LHW programmes. This review focuses specifically on how health information and support is individually tailored in effective programmes by health workers who have, in comparison to health professionals, little formal training.

This review will be of value to those involved in the design, development or implementation of lay health worker interventions as we expect the results to aid the optimisation of such programmes.

### Dissemination of findings

This review will be reported in line with PRISMA guidelines [25]. The findings will be disseminated to the Executive Committee of the Childsmile programme (Scotland’s national oral health improvement programme for children). This is intended to support optimisation of programme delivery. Additionally, the findings will be submitted to a relevant peer-reviewed journal, listed in an online repository for published papers at the University of Glasgow and form part of a thesis for a PhD degree.

## Abbreviations

CINAHL, Cumulative Index of Nursing and Allied Health Literature; EMBASE, Excerpta Medica Database; LHW, lay health worker; MEDLINE, Medical Literature Analysis and Retrieval System Online; MeSH, Medical Subject Headings; PRISMA, Preferred Reporting Items for Systematic Reviews and Meta-analyses; PsycINFO, Psychological Information Database

## Additional files

Additional file 1: The PRISMA-P 2015 checklist for systematic review protocols has been completed and uploaded. (DOC 82 kb) Additional file 2: Inclusion and exclusion criteria used for assessing relevance of studies to be included in the review. (DOC 40 kb) Additional file 3: A sample EMBASE search strategy (which will be adapted for other databases). (DOC 25 kb) Additional file 4: Data extraction form—details of information to be extracted from included studies. (DOC 34 kb)
