# Supplementary material for: How lay health workers tailor in effective health behaviour change interventions: a protocol for a systematic review
Source: Syst Rev. 2016 Jun 16;5:102. doi: 10.1186/s13643-016-0271-z (PMC4910188; doi:10.1186/s13643-016-0271-z)
Supplement: Additional file 1: — The PRISMA-P 2015 checklist for systematic review protocols has been completed and uploaded. (DOC 82 kb) [file 13643_2016_271_MOESM1_ESM.doc]

**PRISMA-P (Preferred Reporting Items for Systematic review and Meta-Analysis Protocols) 2015 checklist: recommended items to address in a systematic review protocol***

| Section and topic | Item No | Checklist item | Reported |
| --- | --- | --- | --- |
| ADMINISTRATIVE INFORMATION | | |  |
| Title: |  |  |  |
| Identification | 1a | Identify the report as a protocol of a systematic review | Report identified as a protocol of a systematic review |
| Update | 1b | If the protocol is for an update of a previous systematic review, identify as such | This protocol is not an update of a previous systematic review |
| Registration | 2 | If registered, provide the name of the registry (such as PROSPERO) and registration number | PROSPERO- CRD42015030071 |
| Authors: |  |  |  |
| Contact | 3a | Provide name, institutional affiliation, e-mail address of all protocol authors; provide physical mailing address of corresponding author | Name, institutional affiliation and e-mail address of all authors provided on title page. Physical mailing address of corresponding author also given. |
| Contributions | 3b | Describe contributions of protocol authors and identify the guarantor of the review | Contributions of protocol authors are indicated throughout the manuscript. |
| Amendments | 4 | If the protocol represents an amendment of a previously completed or published protocol, identify as such and list changes; otherwise, state plan for documenting important protocol amendments | A record of protocol amendments will be maintained by the corresponding author. |
| Support: |  |  |  |
| Sources | 5a | Indicate sources of financial or other support for the review | As described in manuscript, funding came from the Scottish Government through the Childsmile programme. |
| Sponsor | 5b | Provide name for the review funder and/or sponsor | Childsmile. |
| Role of sponsor or funder | 5c | Describe roles of funder(s), sponsor(s), and/or institution(s), if any, in developing the protocol | The Childsmile programme took no direct role in developing the protocol. Members of the Childsmile Evaluation and Research Team agreed the review research questions and guided the review process. |
| INTRODUCTION | | |  |
| Rationale | 6 | Describe the rationale for the review in the context of what is already known | Detailed in manuscript- ‘Background’ section |
| Objectives | 7 | Provide an explicit statement of the question(s) the review will address with reference to participants, interventions, comparators, and outcomes (PICO) | Detailed in manuscript- ‘Aims/Objectives’ |
| METHODS | | |  |
| Eligibility criteria | 8 | Specify the study characteristics (such as PICO, study design, setting, time frame) and report characteristics (such as years considered, language, publication status) to be used as criteria for eligibility for the review | Detailed in manuscript- ‘Method: Eligibility criteria’ and draft of inclusion/exclusion criteria attached as Additional File 2 |
| Information sources | 9 | Describe all intended information sources (such as electronic databases, contact with study authors, trial registers or other grey literature sources) with planned dates of coverage | Detailed in manuscript- ‘Method: Information sources’ |
| Search strategy | 10 | Present draft of search strategy to be used for at least one electronic database, including planned limits, such that it could be repeated | Detailed in manuscript (‘Method: Search strategy’) and draft of strategy attached as Additional File 3 |
| Study records: |  |  |  |
| Data management | 11a | Describe the mechanism(s) that will be used to manage records and data throughout the review | Detailed in manuscript- ‘Method: Data management and selection process’ |
| Selection process | 11b | State the process that will be used for selecting studies (such as two independent reviewers) through each phase of the review (that is, screening, eligibility and inclusion in meta-analysis) | Detailed in manuscript- ‘Method: Data management and selection process’ |
| Data collection process | 11c | Describe planned method of extracting data from reports (such as piloting forms, done independently, in duplicate), any processes for obtaining and confirming data from investigators | Detailed in manuscript- ‘Method: Data extraction’ |
| Data items | 12 | List and define all variables for which data will be sought (such as PICO items, funding sources), any pre-planned data assumptions and simplifications | Detailed in manuscript (‘Method: Data extraction’) and draft of strategy attached as Additional File 4 |
| Outcomes and prioritization | 13 | List and define all outcomes for which data will be sought, including prioritization of main and additional outcomes, with rationale | Detailed in manuscript- ‘Method: Data extraction’ |
| Risk of bias in individual studies | 14 | Describe anticipated methods for assessing risk of bias of individual studies, including whether this will be done at the outcome or study level, or both; state how this information will be used in data synthesis | Detailed in manuscript- ‘Method: Quality assessment’ |
| Data synthesis | 15a | Describe criteria under which study data will be quantitatively synthesised | Detailed in manuscript- ‘Method: Data synthesis’ |
|  | 15b | If data are appropriate for quantitative synthesis, describe planned summary measures, methods of handling data and methods of combining data from studies, including any planned exploration of consistency (such as I^2^, Kendall’s τ) | N/A |
|  | 15c | Describe any proposed additional analyses (such as sensitivity or subgroup analyses, meta-regression) | N/A |
|  | 15d | If quantitative synthesis is not appropriate, describe the type of summary planned | Detailed in manuscript- ‘Method: Data synthesis’ |
| Meta-bias(es) | 16 | Specify any planned assessment of meta-bias(es) (such as publication bias across studies, selective reporting within studies) | Assessed after risk of bias and quality assessment completed for clusters of studies. |
| Confidence in cumulative evidence | 17 | Describe how the strength of the body of evidence will be assessed (such as GRADE) | Detailed in manuscript- ‘Method: Quality assessment’ |

*** It is strongly recommended that this checklist be read in conjunction with the PRISMA-P Explanation and Elaboration (cite when available) for important clarification on the items. Amendments to a review protocol should be tracked and dated. The copyright for PRISMA-P (including checklist) is held by the PRISMA-P Group and is distributed under a Creative Commons Attribution Licence 4.0.**

*From: Shamseer L, Moher D, Clarke M, Ghersi D, Liberati A, Petticrew M, Shekelle P, Stewart L, PRISMA-P Group. Preferred reporting items for systematic review and meta-analysis protocols (PRISMA-P) 2015: elaboration and explanation. BMJ. 2015 Jan 2;349(jan02 1):g7647.*
