# Supplementary material for: How lay health workers tailor in effective health behaviour change interventions: a protocol for a systematic review
Source: Syst Rev. 2016 Jun 16;5:102. doi: 10.1186/s13643-016-0271-z (PMC4910188; doi:10.1186/s13643-016-0271-z)
Supplement: Additional file 2: — Inclusion and exclusion criteria used for assessing relevance of studies to be included in the review. (DOC 40 kb) [file 13643_2016_271_MOESM2_ESM.doc]

**Additional File 2**: Inclusion/exclusion criteria

| **Inclusion** |
| --- |
| 1. The study must report an evaluation of a health behaviour change intervention. |
| 1. A LHW (or multiple LHWs) must be the key individual delivering the intervention. |
| 1. Include interventions where a LHW is delivering the intervention to their own family ONLY as part of delivering the intervention to a wider network (friends/colleagues) or community. |
| 1. Include interventions delivered to all children and all adults, but can be delivered to parents/carers to change behaviour in their children. |
| 1. The outcome of the intervention should be a change in health behaviour (excluding disease management- e.g. diabetes management). This may be the secondary or tertiary outcome (e.g. where the primary outcome may be a change in health status/health measure). |
| 1. The intervention must allow for communication (back and forth) between an individual and LHW(s)   The following methods of delivery can be included:   - Face to face - Telephone   The following methods of delivery can be included IF there is an exchange between an individual and LHW(s):   - Email - Forum - Text |
| 1. There must be evidence that the intervention is tailored (i.e. one or all of the following must be based on an individual assessment of needs/characteristics: the content/context/frames/channel for delivery of the intervention). |
| 1. Individual and group interventions are included as long as there is evidence that an individual assessment of needs/characteristics has been conducted. |
| 1. Interventions taking place in all contexts/settings are included (e.g. community, clinical). Include interventions taking place in contexts similar to the UK (i.e. Western Europe, North America, Australia and New Zealand). |
| **Exclusion** |
| 1. Exclude interventions that are solely delivered by health professionals (people with a formal health profession qualification or degree- e.g. hygienist therapist/nurse/social worker/doctor would be excluded), or those in training for a professional qualification (e.g. medical students). |
| 1. Exclude interventions where lay workers have an exclusively administrative role. |
| 1. Exclude interventions where a LHW is delivering the intervention to their immediate family only. |
| 1. Exclude interventions that do not involve interpersonal communication (e.g. service user interacting with tailored computer program). |
| 1. Exclude interventions that are not tailored |
| 1. Exclude interventions where the outcome is not a change in health behaviour or where the outcome is disease management (e.g. diabetes management). |
| 1. Exclude papers that do not report an evaluated intervention outcome. |
| 1. Exclude interventions taking place outside of contexts similar to the UK (i.e. anywhere outside of Western Europe, North America, Australia and New Zealand). |
