# Supplementary material for: How lay health workers tailor in effective health behaviour change interventions: a protocol for a systematic review
Source: Syst Rev. 2016 Jun 16;5:102. doi: 10.1186/s13643-016-0271-z (PMC4910188; doi:10.1186/s13643-016-0271-z)
Supplement: Additional file 3: — A sample EMBASE search strategy (which will be adapted for other databases). (DOC 25 kb) [file 13643_2016_271_MOESM3_ESM.doc]

**Additional File 3: Search strategy- EMBASE**

1. mentor*.mp
2. (community adj3 worker* or aide*)).mp
3. paraprofessional*.mp
4. community health worker*.mp
5. (support adj3 worker*).mp
6. (social adj3 (assistant* or support) adj3 worker*)).mp
7. community health advisor*.mp
8. (linkworker* or (link adj1 worker*().mp
9. (health adj3 trainer*).mp
10. (home adj2 visit*).mp
11. exp United States/
12. exp Western Europe/
13. exp Australia/
14. exp New Zealand/
15. exp Canada/
16. 11 or 12 or 13 or 14 or 15
17. health promotion.mp or exp health promotion/
18. (behaviour adj1 change)/mp
19. hard-to-reach.mp
20. (deprivation or deprived).mp
21. marginali*.mp
22. underserved.mp
23. disadvantaged.mp
24. health inequal*.mp
25. health dispar*.mp
26. health visit*.mp
27. counselling.mp or exp counselling/
28. counselled.mp
29. (face-to-face adj3 intervention).mp
30. tailor*.mp
31. personalised.mp
32. personalized.mp
33. personalising.mp
34. personalizing.mp
35. personalise.mp
36. personalize.mp
37. individualise.mp
38. individualize.mp
39. individualised.mp
40. inidividualized.mp
41. individualizing.mp
42. individualising.mp
43. (programme adj3 evaluation).mp
44. (service adj3 evaluation).mp
45. Qualitative research.mp or exp qualitative research/
46. RCT.mp
47. exp “randomized controlled trial (topic)”/
48. exp evaluation study/
49. (cluster adj2 randomised).mp
50. (longitudinal adj4 (evaluation* or stud*)).mp
51. (cohort adj4 study).mp
52. exp treatment outcome/
53. exp exploratory research/or exploratory study.mp
54. (process adj2 evaluation).mp
55. trial*.mp
56. ((worker* or advisor* or support* or helper* or influencer*) adj3 (voluntary or volunteer* or lay or peer*)).mp
57. 1 or 2 or 3 or 4 or 5 or 6 or 7 or 8 or 9 or 10 or 56
58. 43 or 44 or 45 or 46 or 47 or 48 or 49 or 50 or 51 or 52 or 53 or 54 or 55 or 56
59. 17 or 18 or 19 or 20 or 21 or 22 or 23 or 24 or 25 or 26 or 27 or 28 or 29 or 30 or 31 or 32 or 33 or 34 or 35 or 36 or 37 or 38 or 39 or 40 or 41 or 42
60. 16 and 57 and 58 and 59
61. Limit 61 to (human and english language)
