# Supplementary material for: How lay health workers tailor in effective health behaviour change interventions: a protocol for a systematic review
Source: Syst Rev. 2016 Jun 16;5:102. doi: 10.1186/s13643-016-0271-z (PMC4910188; doi:10.1186/s13643-016-0271-z)
Supplement: Additional file 4: — Data extraction form—details of information to be extracted from included studies. (DOC 34 kb) [file 13643_2016_271_MOESM4_ESM.doc]

**Additional File 4: Data Extraction Form**

| User ID | Date | Intervention reference | Peer reviewed papers  (+codes) | Additional materials (+ codes) | Papers to be assessed for risk of bias |
| --- | --- | --- | --- | --- | --- |
|  |  |  |  |  |  |

| Peer reviewed papers | | |
| --- | --- | --- |
| Study code | Study method | Result supporting effectiveness/no effect |
|  |  |  |
|  |  |  |
|  |  |  |

| Type of LHW | LHW training | Intervention location | Participant description | Target health behaviour | Theoretical foundation | How theory informed design |
| --- | --- | --- | --- | --- | --- | --- |
|  |  |  |  |  |  |  |

| Assessment: Variables used to determine LHW approach | Type of tailored LHW actions | Specific example of type of tailored LHW actions |
| --- | --- | --- |
|  |  |  |

| Algorithms used | Context | Channel | Format | Dosage | Schedule of assessment and implementation |
| --- | --- | --- | --- | --- | --- |
|  |  |  |  |  |  |
